# Plurigin Ovules and Plurigin Solution in the Treatment of Vulvovaginal Atrophy in Menopausal Women: A Retrospective Monocentric Observational Study

**DOI:** 10.3390/medicina59061108

**Published:** 2023-06-07

**Authors:** Daniele Langella

**Affiliations:** Unità Operativa Materno Infantile, Distretto 57 ASL Napoli 3 Sud, 80059 Torre del Greco, Italy; danilang60@gmail.com

**Keywords:** vulvovaginal atrophy, menopause, sexual function, medical devices, gynecology, non-hormonal therapies

## Abstract

*Background and Objectives*: Vulvovaginal atrophy (VVA) is a condition that affects a large number of women and can significantly impact their quality of life. While several treatments are currently available for VVA, there are potential risks associated with their use. Non-hormonal medical devices have been developed to treat VVA, offering a potential alternative to traditional hormone-based therapies. This study aimed to evaluate the safety and efficacy of the combined treatment with two medical devices, Plurigin Ovules and Plurigin Solution, used as an adjuvant in the treatment of VVA. *Materials and Methods*: This is a retrospective, observational study. Data were collected from medical records of all patients who received the combination treatment of both medical devices as part of normal clinical practice for the treatment of VVA. The performance of the medical devices was analyzed using the THIN Prep. A comprehensive physical examination and gynecological evaluation were conducted before the initiation of treatment (day 0), as well as at follow-up 1 (day 90), follow-up 2 (day 180), and follow-up 3 (day 270). Data analysis included descriptive analysis and statistical tests. *Results*: The study included 76 women (mean age: 59 years). At follow-up 3, 61% of the respondents demonstrated improved THIN Prep results and symptom resolution (*p* < 0.001; CI [0.5003, 0.7197]). Moreover, the percentage of patients reporting dyspareunia, burning, and irritation decreased over the course of the study, with the majority of patients reporting no symptoms at follow-up 3. *Conclusions*: The use of Plurigin Ovules plus Plurigin Solution may be an effective treatment option for VVA, improving vaginal health, alleviating symptoms, and improving sexual function, leading to improved quality of life for women suffering from this condition. However, the study has limitations, such as its retrospective nature, and further studies are needed to confirm the efficacy and safety of these devices.

## 1. Introduction

Vulvovaginal atrophy (VVA), also known as atrophic vaginitis, is a common condition that affects women, particularly those who have reached menopause or undergone certain medical treatments such as chemotherapy or radiation therapy [1,2]. It is characterized by thinning, drying, and inflammation of the vaginal walls, often leading to symptoms such as itching, burning, pain during sexual intercourse (dyspareunia), dysuria, blood loss, and vaginal dryness [3]. VVA is primarily caused by a decline in estrogen levels, which can occur as a natural part of aging or due to certain medical conditions or treatments [1]. Estrogen plays a crucial role in maintaining the thickness and elasticity of the vaginal walls and supporting the growth of healthy bacteria in the vagina, known as *lactobacilli* [4]. When estrogen levels decrease, the vaginal walls become thinner, drier, and more easily irritated, and the balance of healthy bacteria is disrupted [1,4].

The symptoms of vulvovaginal atrophy can significantly impact a woman’s quality of life, affecting her sexual function, relationships, and overall well-being. In 2012, an expert panel termed VVA with its associated symptoms, genitourinary syndrome of menopause (GSM) [5]. While some vasomotor symptoms of menopause may diminish over time, GSM is unlikely to resolve spontaneously and often progresses if left untreated. The choice of therapy for GSM depends on symptom severity, treatment effectiveness and safety, and patient preference.

There are several treatment options, including non-hormonal and hormonal therapies [6]. Non-hormonal therapies include over-the-counter vaginal moisturizers and lubricants, which can help relieve symptoms of dryness and discomfort during sexual intercourse [3,6]. Additionally, regular sexual activity or the use of vaginal dilators can help maintain the elasticity of the vaginal walls [7]. Hormonal therapies, such as vaginal estrogen creams, tablets, or rings, are effective in treating vulvovaginal atrophy by restoring estrogen levels in the vaginal tissues [1]. These treatments have been shown to improve vaginal dryness, itching, and burning, as well as increase sexual satisfaction and reduce the risk of urinary tract infections. While hormonal therapies have been shown to be effective and safe, there are some potential risks associated with their use [8]. Estrogen therapy may increase the risk of certain health conditions, such as breast cancer, stroke, and blood clots, particularly in women who have a history of these conditions [8]. For this reason, there is a need for new effective treatments with fewer side effects for VVA.

Plurigin Ovules and Plurigin Solutions (Praevenio Pharma, Aversa, Italy) are medical devices that have received the European Conformity mark and have been available on the European market since 2019. Plurigin Ovules are non-sterile, disposable medical devices that promote the healing process in inflammatory and dystrophic diseases of the vaginal mucosa. Plurigin Solution is a class I Medical device for vaginal use, indicated as an adjuvant treatment in inflammatory, atrophic, and dystrophic diseases of the vaginal mucosa, including cervicitis and ectropion. Plurigin Ovules and Plurigin Solutions have never been tested in combination for the treatment of VVA.

Therefore, the aim of the present study is to evaluate the performance and safety of the combined use of Plurigin Ovules and Solution in the treatment of VVA. This study’s findings could potentially lead to a new treatment option for VVA that is effective and safe, with fewer side effects than current treatments.

## 2. Materials and Methods

### 2.1. Study Design

This is an investigator-initiated, monocentric, retrospective, observational study to evaluate the safety and efficacy of combined treatment with two medical devices called Plurigin Ovules and Plurigin Solution, used as an adjuvant in the treatment of altered vaginal epithelium associated with bacterial or fungal infections in menopausal women. The retrospective analysis was conducted on patients treated between December 2020 and December 2022 at the Maternal and Child Health Operating Unit, District 57, ASL Napoli 3 Sud, Torre del Greco, 80059, Italy. The study was approved by the Campania SUD ethic committee, protocol number 0017747 of 26 January 2023. Due to the retrospective nature of the study and the prolonged treatment period of the patients, an exemption from the requirement of obtaining informed consent was requested and subsequently approved by the ethics committee. All procedures adhered to the ethical standards set forth by the responsible committee on human experimentation (both institutional and national), as well as the Helsinki Declaration of 1975, as revised in 2008.

### 2.2. Study Population

The study population consisted of adult women who received the combined treatment of the two devices as normal clinical practice for the treatment of VVA. Patients with incomplete medical records were excluded.

Inclusion criteria were diagnosis of atrophy of the vaginal epithelium on cytological examination (made by means of THIN Prep) also associated with bacterial or fungal infections, and menopausal patients. The diagnosis of menopause was established based on the following criteria: presence of amenorrhea for a continuous period of 12 months; confirmation of menopause through hormonal assays, including follicle-stimulating hormone (FSH), luteinizing hormone (LH), and prolactin levels; additional verification by examining thyroid hormone levels.

Exclusion criteria were patients undergoing cervical surgery or with total hysterectomy, autoimmune diseases or patients being treated with immunosuppressants, patients with a history of genitourinary malignancies or abnormal genital bleeding, use of vaginal contraceptives, and pregnancy or breastfeeding.

### 2.3. Data Collection

Data were collected from medical records of all patients who received a combination treatment of both medical devices. The treatment regimen was as follows: every month, the patient underwent a vaginal wash with Plurigin Solution, followed by the administration of Plurigin Ovules at night for 10 days, followed by another vaginal wash with Plurigin Solution. Then, they stopped and repeated the same treatment program the following month. This regimen was repeated for a total of 9 months.

The performance of the medical devices was analyzed by using the THIN Prep (Hologic Italia Srl., Rome, Italy), performed by the Pathology Unit, ASL Napoli 3 SUD. The THIN Prep is a liquid-based pap testing intended for use in the screening and detection of cervical cancer, pre-cancerous lesions, atypical cells, and all other cytologic categories as defined by the Bethesda system for reporting results of cervical cytology [9]. THIN Prep results give information on Human Papilloma Virus (HPV), fungal and bacterial infections, and the cytological state of the epithelium, evaluated as atrophic, ipotrophic, normal. All treatments given in addition to the investigational devices and all previous treatments given within 7 days before day 0 of the investigation were recorded, together with the indication, quantity, or dose administered, and the dates and time of administration. Demographic information, medical history, weight measurements, adverse events, and clinical outcomes were also collected. Each subject was followed up for 9 months (follow-up 3, day 270). A comprehensive physical examination and a complete gynecological evaluation were conducted before the initiation of treatment (day 0), as well as at 3 (follow-up 1, day 90), 6 (follow-up 2, day 180), and 9 months, which included measurements of height, weight, symptom collection, pelvic examination, breast examination, and cytological examination. An overview of all assessments is provided in Table 1.

### 2.4. Clinical Investigation Endpoints

All complications and outcomes were reviewed from patients’ medical records.

Primary efficacy endpoint: Percentage of responders after 9 months of treatment. Responders are identified by an improvement of the THIN Prep on the epithelium state (atrophic, ipotrophic, or normal) and reduction in symptoms, such as dyspareunia and burning. 

Secondary efficacy endpoints: Percentage of responders after 3 and 6 months of treatment. Improvement of the score of symptoms associated with vaginal atrophy, such as dyspareunia, vaginal dryness at 3 and 6 months. 

Safety endpoint: Adverse events (AEs). AEs were systematically monitored during each follow-up visit, which occurred every 3 months. The monitoring process involved a comprehensive objective examination that encompassed gynecologic examination with supportive transvaginal ultrasound; additionally, blood or biological laboratory tests were conducted as deemed necessary. For the safety analysis, AEs were coded with the Medical Dictionary for Regulatory Activities (MedDRA) version 16.0 and summarized by system organ class and preferred term. Adverse device effects and serious adverse events (SADEs), as well as adverse events leading to withdrawal, were summarized separately.

### 2.5. Medical Devices

Plurigin Ovules is a non-sterile, disposable medical device intended to promote reparative processes in inflammatory and dystrophic diseases of the vaginal mucosa. It is also indicated as an adjuvant in healing processes and as a moisturizer in cases of vaginal dryness. The supportive function in the processes of re-epithelialization and hydration can be attributed to both gelatin and glycerin. These two substances form a protective film on the skin surface and retain water to promote the humectant properties of the product. In this way, the two substances protect the mucosa from the external environment, thus indirectly favoring the physiological restoration of tissues and the normalization of damaged surfaces.

Plurigin Solution is a class I medical device for vaginal use. It is indicated as an adjuvant treatment in phlogistic, atrophic, and dystrophic diseases of the vaginal mucosa, such as cervicitis and ectropion. It can also serve as an adjuvant in pre- and post-operative prophylaxis after physical treatment. 

These devices were used in combination in this study.

### 2.6. Data Analysis

The sample size was determined based on the percentage of responders after 9 months of treatment, with a natural remission of about 15% expected and about 35% in the treated groups considered, with a power of 80% (two tailed). Demographic data and other covariates such as weight, medical history, and prior and concomitant medication were analyzed descriptively. The primary efficacy variable was analyzed using a one-proportion z-test and chi-squared test with a 2-sided significance level of 5%. The test statistic used was a z-score (z) defined by the equation z = (p − P)/σ where P is the hypothesized value of the population proportion in the null hypothesis, p is the sample proportion, and σ is the standard deviation of the sampling distribution. The primary efficacy analysis was based on the protocol population and was considered confirmatory. Descriptive statistics were also provided. The secondary efficacy variables were summarized in tables and shift tables where appropriate, using descriptive statistics. Additionally, box plots and bar charts were used for graphic illustration of results.

## 3. Results

### 3.1. Patients

**Figure 1:**
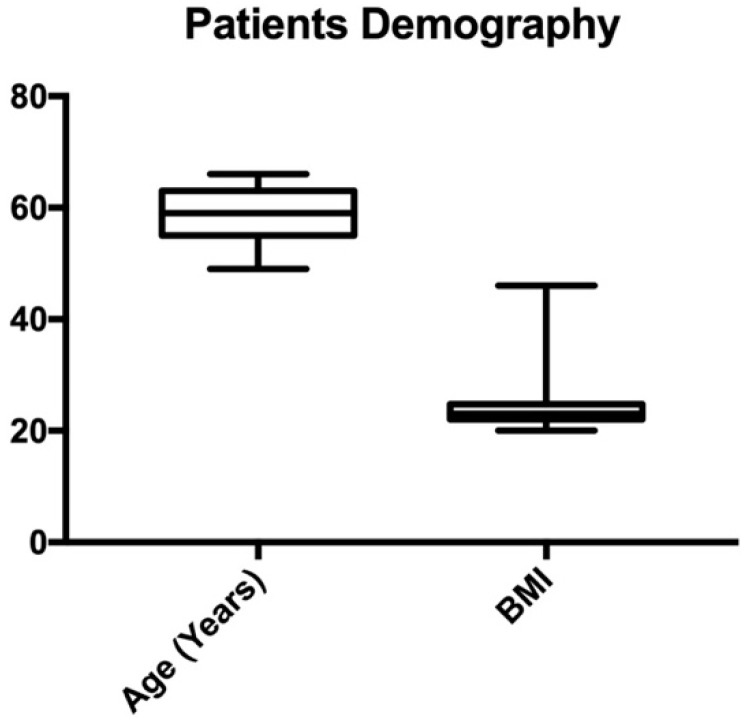
Patients demography, with age and body mass index (BMI).

Seventy-seven subjects met the inclusion criteria and were included in this retrospective study. All the subjects in the study were Caucasian female. The median age was 59 years (range 49–66) (Table 2). None of the patients included had a significant medical history. Out of the 76 patients who received treatment with the investigational devices, 41 (32.0%) had previously attempted to use other devices for the same condition. However, in order to participate in the study, these treatments had to be discontinued for at least 15 days prior to starting the investigational devices. Demography details are shown in Figure 1.

### 3.2. Baseline Assessment of Vaginal Epithelium and Symptoms

**Figure 2:**
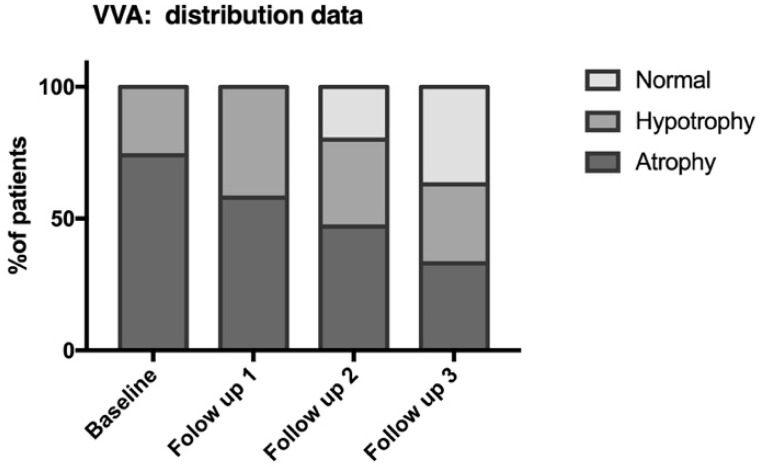
An assessment of the vaginal epithelium of N = 76 patients was performed using THIN Prep at baseline and follow-ups 1, 2, and 3. According to THIN Prep results, the vaginal epithelium was classified as normal, hypotrophy, and atrophy. Overtime, there is a significant improvement of patients’ epithelial state with many patients with atrophic epithelium transitioning to hypotrophic or normal and many patients with hypotrofic epithelium at baseline transitioning to normal. All *p* < 0.005 (as reported in the text).

All patients included in the protocol had a diagnosis of VVA. As assed by the THIN Prep, at day 0 of the treatment, 74% of patients had an atrophic vaginal epithelium, 26% a hypotrophic epithelium, and 0% a normal epithelium (Figure 2). The reported symptoms were burning (45%), dyspaneuria (43%), dysuria (37%), irritation (32%), hitching (32%), and blood loss (16%) (Table 3).

### 3.3. Efficacy

**Figure 3:**
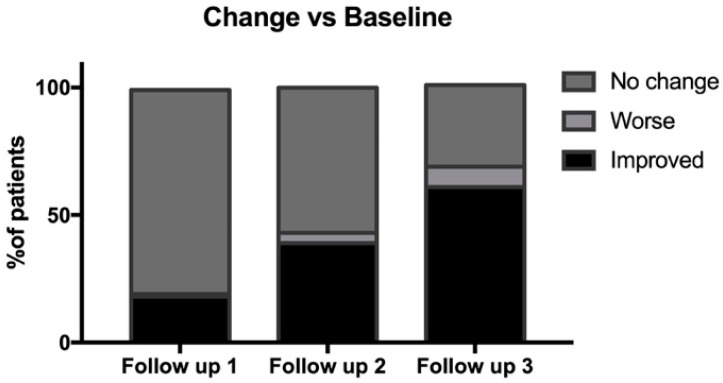
A comparison of the vaginal epithelium at follow-up 1, follow-up 2, and follow-up 3 to the baseline, as evaluated through THIN PREP. The epithelium was categorized into three groups: no change, if the results of the THIN Prep and the symptoms were similar to baseline; worse, defined as those who demonstrated worsened THIN Prep and no symptom resolution; improved, defined as those who demonstrated improved THIN Prep results and symptom resolution. The percentage of responders (improved) compared to baseline was statistically significant at all follow-ups. *p* < 0.001.

The study’s primary efficacy endpoint was the change in THIN Prep results from day 0 to day 270. A statistically significant improvement in THIN Prep results was observed at follow-up 3 (day 270) compared to baseline (Figure 3). As previously reported, at baseline, 74% of patients had atrophic epithelium, 26% had hypotrophic epithelium, and 0% had normal epithelium (Table 3). At follow-up 3 (270 days), 33% of patients still had atrophy, 30% had hypotrophy, and 37% had a normalized epithelium (Table 3). The percentage of responders, defined as those who demonstrated improved THIN Prep results and symptom resolution, was 61% at follow-up 3 (270 days) (Figure 3). These data were analyzed using a one-proportion z-test, resulting in a z-statistic of 11.23077 and a *p*-value of less than 0.001, with a 95% confidence interval of [0.5003, 0.7197]. These results confirm the statistically significant performance of the investigational device after 9 months of treatment. The secondary efficacy endpoints of this study included the percentage of patients classified as responders after 3 and 6 months of treatment. The results are presented in Table 4, which reports the percentage of patients who experienced improvement, worsening, or no change in symptoms at follow-up 1, follow-up 2, and follow-up 3. At follow-up 1, 18% of patients showed improvement, 1% worsened, and 80% demonstrated no change in symptoms. At follow-up 2, 39% of patients showed improvement, 4% worsened, and 57% had no change in symptoms.

### 3.4. Symptoms

**Figure 4:**
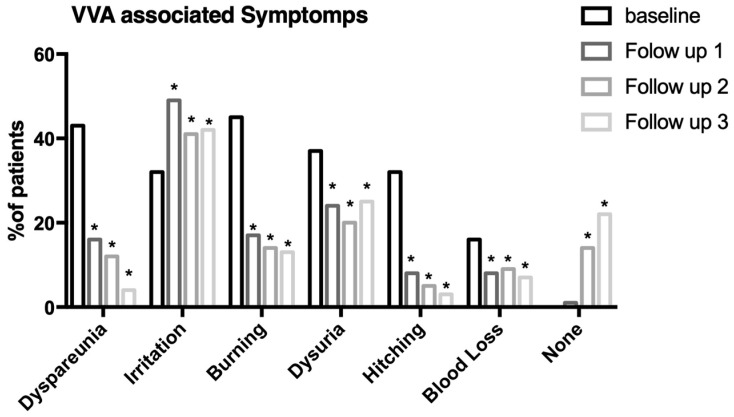
The percentage of patients who reported experiencing symptoms at baseline and at three follow-up intervals: follow-up 1, follow-up 2, and follow-up 3. * *p* < 0.001.

Table 4 presents the percentage of patients with various symptoms at different time points throughout the study. At baseline, the most common symptoms reported were burning (45%), dyspareunia (43%), and irritation (32%) (Figure 4). At follow-up 1, there was a significant reduction in the percentage of patients reporting dyspareunia (16%) and burning (17%), but an increase in the percentage of patients reporting irritation (49%) (all *p* < 0.001; Figure 4). At follow-up 2, the percentage of patients reporting symptoms continued to decrease for most symptoms, with the exception of irritation (41%) which remained high (Figure 4). At follow-up 3, the percentage of patients reporting symptoms decreased even further, with only a small percentage reporting dyspareunia (4%), burning (13%), and irritation (42%) (all *p* < 0.001; Figure 4). The majority of patients reported no symptoms at follow-up 3 (22%) (Figure 4).

### 3.5. Adverse Events

No adverse events related with the investigational devices were reported.

## 4. Discussion

VVA is a condition that affects many women, especially those who are postmenopausal [6]. This condition is associated with several symptoms that can significantly impact their quality of life, such as vaginal dryness, burning, itching, and painful intercourse [2,10]. Dyspareunia or painful intercourse can be particularly distressing for women and can negatively affect their sexual function, leading to decreased sexual desire, arousal, and satisfaction [11,12]. While several treatments are available for vaginal atrophy, there is still a need for new and more effective therapies. Recently, new non-hormonal medical devices have been developed to treat VVA, offering a potential alternative to traditional hormone-based therapies [13,14].

This retrospective study evaluated the efficacy of the combination of Plurigin Ovules and Plurigin Solution for the treatment of VVA in a population of 76 Caucasian women. The results showed a statistically significant improvement in THIN Prep results from day 0 to day 270, indicating that the investigational devices were effective in improving vaginal health over the course of the study (Figure 3). Furthermore, the percentage of responders who demonstrated improved THIN Prep results and symptom resolution was 61% at follow-up 3 (270 days) (Table 4). This suggests that the investigational devices not only improved vaginal health but also helped to alleviate the symptoms associated with VVA. Specifically, the percentage of patients reporting dyspareunia, burning, and irritation decreased over the course of the study, with the majority of patients reporting no symptoms at follow-up 3 (Figure 4). Moreover, at follow-up 1 (90 days), only a small percentage (18%) of patients showed improvement, whereas the majority (80%) demonstrated no change in symptoms (Table 3). However, at follow-up 2, a larger proportion of patients (39%) showed improvement, and the percentage of patients with no change in symptoms decreased to 57% (Table 3). This suggests that the treatment may have a delayed effect, taking months to show significant improvements in symptoms. It is worth noting that the percentage of patients who worsened was very low at the three follow-up time points, indicating that the treatment is likely safe and well-tolerated (Table 4).

Improvements in vaginal health and symptom relief can have a significant impact on quality of life, particularly with respect to sexual function [7]. Indeed, many women experience sexual problems as a result of vaginal atrophy, including decreased libido, difficulty achieving orgasm, and painful intercourse [7,11,15]. The results of this study suggest that treatment with investigational devices may help to alleviate these sexual problems and improve sexual function, leading to improved quality of life for women with VVA.

As a retrospective study, there are several limitations to consider when interpreting the results. One major limitation is the lack of a control group, which makes it difficult to draw conclusions about the effectiveness of the investigational devices compared to other treatments or no treatment at all. Additionally, the study relied on self-reported symptoms and patient-reported outcomes, which can be subject to bias and may not accurately reflect the true extent of improvement in vaginal health and symptom relief. Finally, the study included a population of 76 Caucasian women, so the results may not be generalizable to other populations or ethnic groups.

## 5. Conclusions

VVA is a common condition that can have a significant impact on quality of life for women, particularly with respect to sexual function. The results of this study suggest that the combined use of Plurigin Ovules plus Plurigin Solution may be an effective treatment option for VVA, improving vaginal health, alleviating symptoms, and improving sexual function, leading to improved quality of life for women suffering from this condition. Overall, while the results suggest that the treatment may have some potential benefits, further research is needed to confirm the findings and determine which patients may be most likely to respond to the treatment. Additionally, future studies should also investigate the long-term effects of the treatment.

## Figures and Tables

**Table 1:** A summary of all the assessments collected during the study at visit 1 (day 0) and at follow-up 1 (visit 2, day 90), follow-up 2 (visit 3, day 180), and follow-up 3 (visit 4, day 270); a: Before application of the investigational device, if applicable.

Assessment	Day 0 Visit 1	Day 90 Visit 2	Day 180 Visit 3	Day 270 Visit 4
Demography	x_a_			
Medical history	x_a_			
Prior medication	x_a_			
Weight measurement	x_a_			
THIN PREP	x_a_	x	x	x
Simptomps recording	x_a_	x	x	x
Concomitant medication	x_a_	x	x	
Adverse event collection	x_a_	x	x	x

**Table 2:** Subject demography (age and BMI). BMI = body mass index; N = number of subjects, STD = standard deviation.

**Age (years)**	Median	59
	Range	49–66
**BMI**	Mean	23.68
	STD	2.9

**Table 3:** The prevalence of symptoms, as reported by patients, over four time points: baseline, follow-up 1, follow-up 2, and follow-up 3.

	Baseline	Folow-Up 1	Follow-Up 2	Follow-Up 3
Dyspareunia	43	16	12	4
Irritation	32	49	41	42
Burning	45	17	14	13
Dysuria	37	24	20	25
Hitching	32	8	5	3
Blood Loss	16	8	9	7
None	0	1	14	22

**Table 4:** The percentage of patients who have either improved, had worse symptoms, or had no change in symptoms in three different follow-up periods.

	Improved	Worse	No Change
Follow-up 1	18	1	80
Follow-up 2	39	4	57
Follow-up 3	61	8	32

## Data Availability

The study’s data is not publicly available due to privacy restrictions. However, interested individuals can obtain the data by sending a specific request to the author.
